# Supplementary material for: T-705-Derived Prodrugs Show High Antiviral Efficacies against a Broad Range of Influenza A Viruses with Synergistic Effects When Combined with Oseltamivir
Source: Pharmaceutics. 2023 Jun 14;15(6):1732. doi: 10.3390/pharmaceutics15061732 (PMC10304372; doi:10.3390/pharmaceutics15061732)
Supplement: Supplementary file 1 [file pharmaceutics-15-01732-s001.zip › pharmaceutics-2385837-supplementary.pdf]

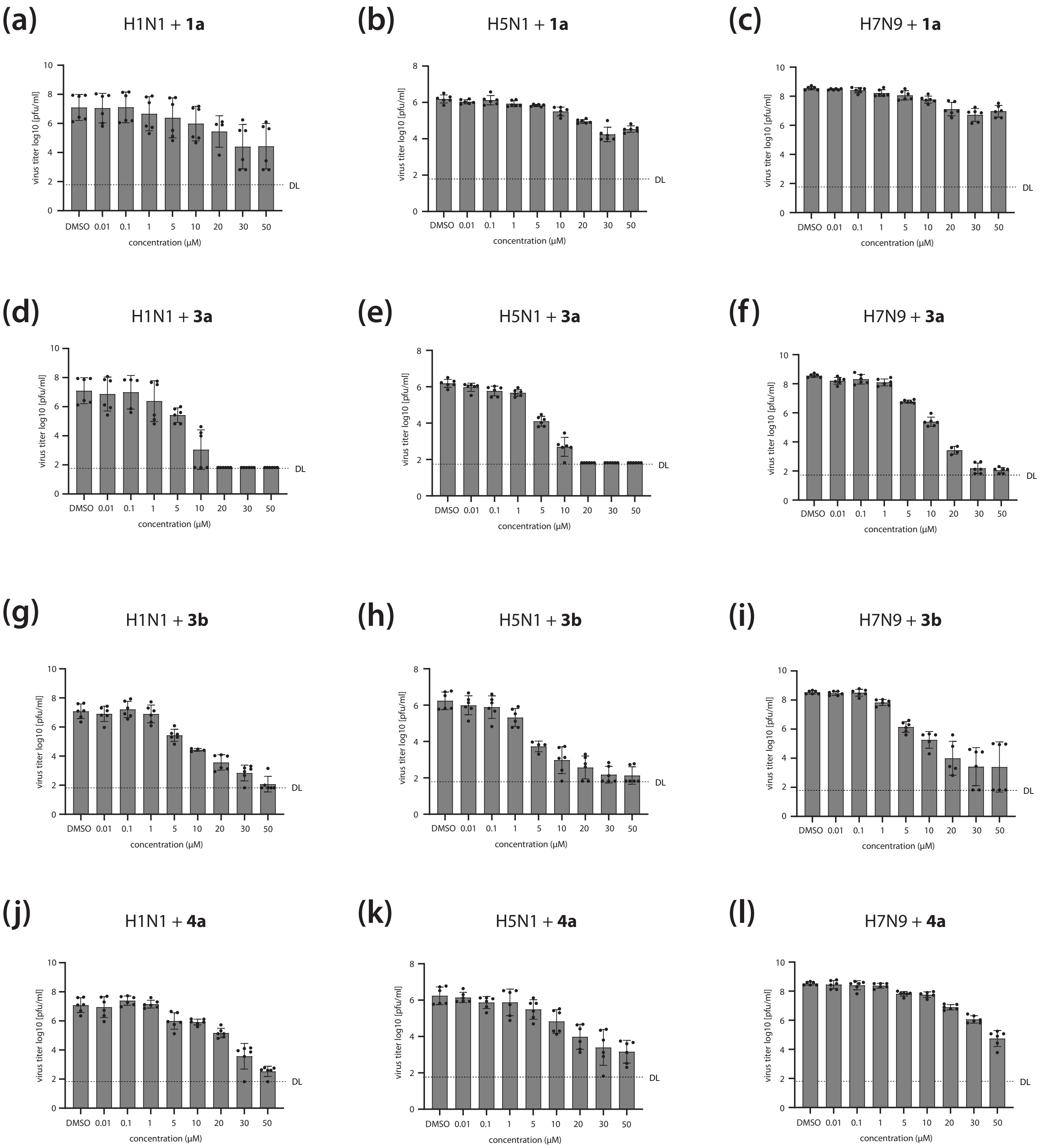
(a)
H1N1 + 1a
(b)
H5N1 + 1a
(c)
H7N9 + 1a
(d)
H1N1 + 3a
(e)
H5N1 + 3a
(f)
H7N9 + 3a
(g)
H1N1 + 3b
(h)
H5N1 + 3b
(i)
H7N9 + 3b
(j)
H1N1 + 4a
(k)
H5N1 + 4a
(l)
H7N9 + 4a
virus titer log10 [pfu/ml]
concentration (µM)
DMSO
0.01
0.1
1
5
10
20
30
50
DL

Figure S1. Antiviral activity of T-705 (1a) and the T-1106 prodrugs (3a, 3b and 4a) against seasonal and avian influenza A viruses. *Related to Figures 2 and 3.*

(**a-l**) MDCKII cells were infected with H1N1 (**a,d,g,j**), H5N1 (**b,e,h,k**) or H7N9 (**c,f,I,l**) IAV at a multiplicity of infection of 0.01 and subsequently treated with T-705 **1a** (**a,b,c**) or its prodrug derivates **3a** (**d,e,f**), **3b** (**g,h,i**) and **4a** (**j,k,l**) at eight different concentrations (range: 0.001 – 50 μM). Cell culture supernatants were collected at 24 h post infection and infectious virus titers were determined by plaque assay. DMSO treatment was used as negative control (no inhibition). Shown are the virus titers as log10 plaque-forming units per ml (pfu/ml) ± SD of two independent biological replicates, each performed with technical triplicates.

Figure S2

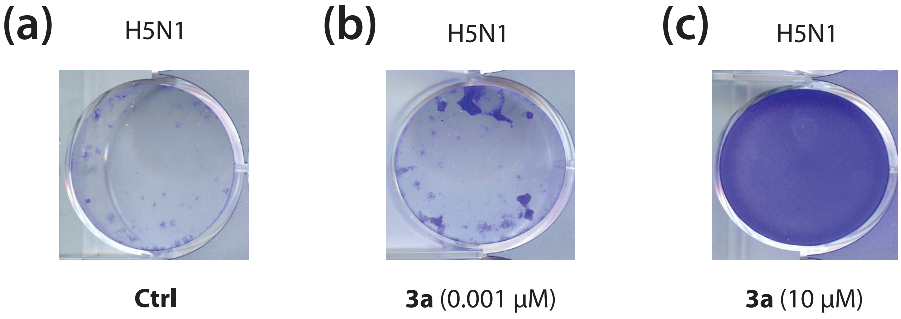

Figure S2. H5N1 plaques on MDCK cells. ***Related to Figure 3.***

(**a-c**) MDCK cells were infected with H5N1 HPAIV (MOI 0.01) and either control-treated (**a**) or treated with DP prodrug **3a** at two different concentrations (**b**, low-dose 0.001 μM; **c**, high-dose 10 μM). Cell culture supernatants were collected at 24 h post infection and titrated by plaque assay using serial dilutions for all conditions. The serial dilution data have been used as a basis to calculate viral titers based on plaque numbers. Shown are representative images at 10^3 dilution.

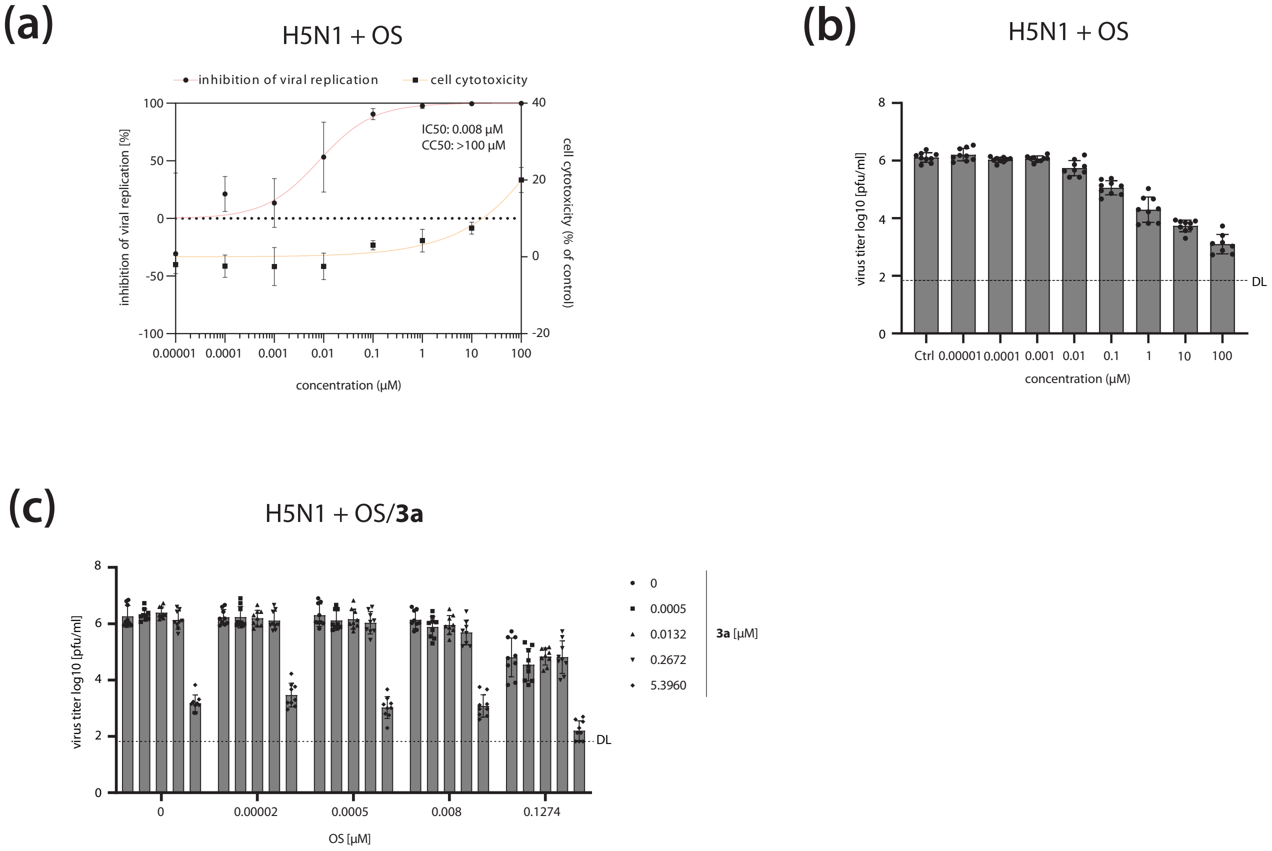
(a)
H5N1 + OS
inhibition of viral replication
cell cytotoxicity
IC50: 0.008 µM
CC50: >100 µM
inhibition of viral replication [%]
cell cytotoxicity (% of control)
concentration (µM)
0.00001 0.0001 0.001 0.01 0.1 1 10 100
(b)
H5N1 + OS
virus titer log10 [pfu/ml]
DL
Ctrl 0.00001 0.0001 0.001 0.01 0.1 1 10 100
concentration (µM)
(c)
H5N1 + OS/3a
virus titer log10 [pfu/ml]
DL
0 0.00002 0.0005 0.008 0.1274
OS [µM]
0
0.0005
0.0132
0.2672
5.3960
3a [µM]

Figure S3. Antiviral activity of T-1106-derived prodrug 3a in combination with the neuraminidase inhibitor oseltamivir (OS) against H5N1 HPAIV replication. *Related to Figure 4.*

(**a,** left y axis) MDCKII cells were infected with H5N1 at a multiplicity of infection of 0.01 and subsequently treated with the neuraminidase inhibitor oseltamivir (OS) at eight different concentrations (range: 0.00001 – 100 μM). Cell culture supernatants were collected at 24 h post infection and infectious virus titers were determined by plaque assay. Mock treatment was used as a negative control. Shown is the average inhibition of viral replication (%) ± SD by the respective compound, compared to the Mock control treatment (0% inhibition). Non-linear regression (red curve) was performed to determine the inhibitory concentration 50 (IC_{50}) value, which is indicated in the upper right corner of the graph (**a,** right y axis) MDCKII cells were treated with neuraminidase inhibitor oseltamivir (OS) at eight different concentrations (range: 0.00001 – 100 μM). At 24 h post treatment, cell viability was measured using a MTT-based assay according to manufacturer's instructions. Mock treatment was used as negative control. Shown is the average reduction in cell viability (i.e. cell cytotoxicity, in %) ± SD, compared to the DMSO control treatment which was arbitrarily set to 0% cell cytotoxicity. Non-linear regression (orange curve) was performed to determine the cell cytotoxicity 50 (CC_{50}) value, which is indicated in the upper right corner of the graph. (**b**) H5N1 HPAIV virus titers as log10 plaque-forming units (pfu/ml) ± SD in MDCKII cells treated with the neuraminidase inhibitor oseltamivir (OS; range 0.00001-100μM) at 24 h post infection (*related to Figure S2a*). Mock treatment was used as negative control (Ctrl) (0% inhibition). The detection limit (DL) is indicated with a dashed line. (**a,b**) Data are derived from three independent biological replicates, each performed in technical triplicates. (**c**) MDCKII cells were infected with H5N1 HPAIV at a multiplicity of infection of 0.01 and subsequently treated with combinations of the T-1106-derived prodrug **3a** and the neuraminidase inhibitor oseltamivir. The tested concentrations are based on the respective IC_1, IC_{10}, IC_{50} and IC_{90} values for each drug. Cell culture supernatants were collected at 24 h post infection and infectious virus titers were determined by plaque assay. DMSO treatment was used as negative control (0% inhibition). Shown are the virus titers as log10 plaque-forming units per ml (pfu/ml) ± SD of three independent biological replicates, each performed with technical triplicates.
